# Reuse Potential of Pacemakers and Implantable Cardioverter-defibrillators - Some Real World Data

**DOI:** 10.1016/s0972-6292(16)30625-8

**Published:** 2013-06-25

**Authors:** SP Abhilash, Narayanan Namboodiri

**Affiliations:** Sree Chitra Tirunal Institute for Medical Sciences and Technology, Trivandrum, India

**Keywords:** Pacemakers, Implantable Cardioverter-defibrillators, Reuse Potential

Patients who survive either ventricular fibrillation or sustained ventricular tachycardia carry a high risk of further episodes, which could be fatal. Anti-arrhythmic drugs were the standard treatment for patients with malignant ventricular arrhythmias, but despite using the best appropriate medical treatment, arrhythmia recurrence rates remain high at 40-50% at five years. Implantable cardioverter defibrillators (ICD) are effective at treating ventricular arrhythmias and there has been clear evidence that they reduce mortality. Cardiac resynchronization therapy (CRT) improves symptoms and reduces morbidity and mortality in patients with advanced heart failure. In addition, many patients have indications for combined ICD and CRT therapy.

While the number of ICD/CRT implantations in the developed world has increased, device utilization rates are less in low- and middle-income countries, despite having higher incidence of risk factors for sudden cardiac death and heart failure. The primary obstacle to the growth of this therapy in developing nations is its cost - ranging from $8000 to $16,000 depending on the model and the features available. Due to its cost, this therapy is currently accessible only to the affluent in developing countries, whereas the subset of patients who requires this therapy is increasing rapidly. According to available data, only a few hundreds of patients in India which has a population of over 120 million receive ICD implants every year, whereas in developed countries with much smaller population the number is at least in thousands.

In this context, devices donated for use by charitable organizations, industry or devices harvested by physicians/industry for reuse, becomes extremely important to patients in low- and middle-income countries. Reuse of devices poses risks such as infection, device malfunction and premature battery depletion. But, recently published studies have shown that there is no increase in morbidity or mortality associated with device reuse, if implanted with good technique and proper sterile precautions. However, there is no proper data or registry available regarding the availability of devices with potential for reuse in the developing or even in the developed world.

The HRS guidelines recommend that physicians seek patients' consent for post-mortem device retrieval while they are alive. In routine practice, informed consent for device implants rarely includes a discussion regarding post-mortem handling of devices. A survey of patient preferences showed that 87% of patients did not know how devices were handled after their death despite the fact that majority were willing to have their devices retrieved for reuse [1]. Moreover, patient preferences for post-mortem handling affect health policies of various governing bodies.

In an interesting study published in this issue, Iyer IR and Mackall J tried to unravel veterans' preferences regarding post-mortem handling of devices and also to create some data on reuse potential of these expensive devices [2]. In this survey, 44.6% of patients wished to donate their devices for post mortem reuse as their first choice and 79% chose this among their top three options.The analysis of database showed that 25.6% of all devices implanted in the deceased cohort had potentially useful remaining battery life.

This is an important study considering that data regarding reuse or reuse potential of these expensive life saving devices are so scarce and its implications especially in developing countries are many. Major limitations of this study are small sample size and retrospective nature for assessing reuse potential of devices. Devices and battery technology analyzed in this study are different from currently used devices; recent generation devices show longer battery life and therefore may offer greater reuse potential. A larger study utilizing large datasets maintained by device manufacturers and multiple centers may give a better estimate of number of devices with significant reuse potential in order to formulate a health policy on this rather important issue.

## References

[R1] Kirkpatrick JN (2007). Postmortem interrogation and retrieval of implantable pacemakers and defibrillators: a survey of morticians and patients. J Cardiovasc Electrophysiol.

[R2] Iyer Indiresha R (2013). Patient Preferences Regarding Device Reuse and Potential of Devices for Reuse - A Study in a Veteran Population. Indian Pacing Electrophysiol J.

